# C-Reactive Protein as a Predictor of Cardiovascular Events and Mortality in Obstructive Sleep Apnea: A Propensity-Matched Analysis Using the TriNetX Research Network

**DOI:** 10.1055/s-0046-1829074

**Published:** 2026-10-09

**Authors:** Ekow Essien, Abena Agyekum, Abraham Carboo, Gloria Amoako, Justice Owusu-Achiaw, Samuel B. Governor

**Affiliations:** 1 Department of Internal Medicine, Advocate Aurora Health Care, Milwaukee, Wisconsin, United States; 2 Department of Medicine, SUNY Downstate Health Sciences University, Brooklyn, New York, United States; 3 Department of Medicine, Yale-Waterbury Hospital, Waterbury, Connecticut, United States; 4 Department of Polyclinic, 37 Military Hospital, Accra, Ghana; 5 Department of Internal Medicine, St. John's Episcopal Hospital, New York, New York, United States

**Keywords:** obstructive sleep apnea, C-reactive protein, Inflammation, cardiovascular disease, mortality

## Abstract

**Introduction:**

Obstructive sleep apnea (OSA) increases cardiovascular disease risk through systemic inflammation, particularly through elevated C-reactive protein (CRP). This study investigated whether CRP stratification predicts mortality and cardiovascular events in OSA patients.

**Methods:**

We conducted a retrospective cohort analysis using the TriNetX Research Network, encompassing electronic health records from 130 healthcare institutions. Adults (ages 18–90 years) with OSA were stratified by CRP concentration: elevated (≥ 3.0 mg/L) versus low (≤ 2.9 mg/L). After propensity score matching for demographics and comorbidities, 240,727 participants per cohort were followed for five years. Primary outcome was all-cause mortality; secondary outcomes included cardiovascular events, arrhythmias, and acute kidney injury.

**Results:**

Elevated CRP was associated with significantly higher all-cause mortality (11.3% versus 7.0%; HR 1.695, 95% CI 1.662–1.727; *p* < 0.001). The elevated CRP group demonstrated heightened cardiovascular risk: cardiogenic shock (1.0% versus 0.5%; HR 2.003), heart failure (9.8% versus 6.5%; HR 1.594), pulmonary hypertension (4.9% versus 3.4%; HR 1.508), and acute kidney injury (11.0% versus 6.4%; HR 1.825), all *p* < 0.001. Arrhythmic complications were also increased: atrial fibrillation/flutter (6.3% versus 4.8%; HR 1.372) and ventricular tachycardia (2.3% versus 1.8%; HR 1.388), both *p* < 0.001.

**Conclusion:**

Elevated CRP constitutes a robust predictor of mortality and cardiovascular morbidity in OSA populations. Assessment of inflammatory biomarkers may enhance risk stratification, informing intensified monitoring and targeted therapeutic interventions.

## Introduction

An estimated 936 million adults worldwide experience obstructive sleep apnea,1 which is characterized by recurrent upper airway collapse during sleep, producing alternating patterns of oxygen deprivation and sleep disruption. 2 While initially recognized primarily for its impact on sleep architecture and daytime alertness, accumulating evidence positions OSA as an independent contributor to cardiovascular pathology, encompassing hypertensive disease, coronary atherosclerosis and rhythm disturbances. 3

OSA contributes to cardiovascular pathology through multiple intersecting mechanisms. Oxygen deprivation activates sympathetic pathways, sustaining blood pressure elevation and altering cardiac rhythm patterns. Concurrent reoxygenation events produce oxidative molecules that injure vascular endothelium, 4 subsequently diminishing normal vessel relaxation and facilitating atherosclerotic progression. 5

Inflammatory biomarkers are consistently elevated in OSA populations compared with matched controls. 6 Among these markers, C-reactive protein has garnered particular attention. Interleukin-6 and related cytokines trigger hepatic CRP synthesis; this protein both indicates and potentially contributes to cardiovascular damage. 7 CRP concentrations correlate with cardiovascular event rates across diverse populations, independent of traditional risk factors. 8 In OSA, CRP levels remain elevated even after adjusting for obesity and metabolic derangements, suggesting disease-specific inflammatory activation. 9

Although CRP correlates with OSA in cross-sectional studies, its prognostic utility remains unclear. 10 Prior research has featured limited sample sizes, brief observation periods, and narrow outcome measures; few investigations have used propensity matching to control confounding adequately. 11

Clarifying the relationship between CRP and clinical outcomes in OSA carries substantial clinical implications. If elevated CRP identifies a high-risk subpopulation among individuals with OSA, this could inform risk-stratified management approaches. High-risk individuals might benefit from intensified cardiovascular surveillance, aggressive risk factor modification, or targeted anti-inflammatory interventions. 12 Conversely, patients with OSA but low inflammatory burden might be managed less aggressively, optimizing resource allocation.

The present study therefore undertook this large-scale investigation using a federated health research platform to comprehensively evaluate associations between CRP concentrations and long-term outcomes in OSA patients. We hypothesized that OSA patients with elevated CRP would demonstrate increased rates of mortality and cardiovascular events compared with those with low CRP concentrations, independent of traditional cardiovascular risk factors.

## Methods

### Data Source

De-identified electronic health records from 130 global healthcare organizations were analyzed through the TriNetX Research Network. This federated platform enables real-time analysis of HIPAA-compliant anonymized records spanning academic centers, community facilities, and integrated systems, supporting broad applicability of findings.

### Study Population

Adult individuals aged 18-90 years with a documented OSA diagnosis, operationalized through ICD-10 code G47.33, were identified. This code encompasses both adult and pediatric OSA; however, age restrictions ensured inclusion of adult patients only. Participants required at least one CRP measurement meeting quality thresholds (within plausible range, temporally associated with OSA diagnosis).

Cohort stratification employed established cardiovascular risk thresholds. The elevated CRP cohort comprised individuals with CRP ≥ 3.0 mg/L ( *n* = 601,398 before matching), while the low CRP cohort included those with CRP ≤ 2.9 mg/L ( *n* = 240,742 before matching). The 3.0 mg/L cut point reflects published cardiovascular risk thresholds 13 : concentrations under 1.0 mg/L suggest low risk, 1.0–3.0 mg/L moderate risk, and over 3.0 mg/L high risk.

The index date for each participant was defined as the first encounter that met both criteria: a documented OSA diagnosis and a qualifying CRP measurement. This approach ensures temporal alignment between exposure (CRP status) and the initiation of outcome surveillance. Participants were followed for up to 1,825 days (five years) from the index date or until censoring (loss to follow-up or end of data availability).

### Outcomes

All-cause mortality was the primary endpoint, assessed via death records in the electronic health record system. Secondary cardiovascular outcomes included: cardiogenic shock (ICD-10 R57.0), heart failure (ICD-10 I50 series including all subtypes), ventricular tachycardia (ICD-10 I47.2 series), atrial fibrillation and flutter (ICD-10 I48), acute kidney injury (ICD-10 N17 series), chronic kidney disease (ICD-10 N18), pulmonary hypertension (ICD-10 I27.2 series), cerebrovascular disease (ICD-10 I60-I69 range), and pulmonary embolism (ICD-10 I26).

Additional outcomes included healthcare utilization (hospital admissions and intensive care unit admissions) and cardiovascular interventions (pacemaker/implantable cardioverter-defibrillator implantation, cardiac catheterization). Outcome ascertainment employed validated ICD-10 and procedural coding algorithms with demonstrated accuracy in administrative health data.

### Propensity Score Matching

To minimize confounding bias, we implemented propensity score matching using a nearest-neighbor algorithm with a 1:1 matching ratio. Propensity scores were calculated using multivariable logistic regression models incorporating demographics (age, sex, race, ethnicity) and baseline comorbidities. Comorbidity adjustment included acute kidney failure, chronic kidney disease, metabolic disorders (including diabetes mellitus, dyslipidemia, obesity), and hypertensive diseases—all recognized OSA-associated conditions and cardiovascular risk factors.

Matching balance was assessed using standardized mean differences, with values < 0.05 indicating adequate balance. This threshold is more stringent than the commonly used < 0.10 criterion, thereby providing greater confidence in confounder control. Following propensity score matching, 240,727 participants remained in each cohort, ensuring identical sample sizes and balanced baseline characteristics.

### Statistical Analysis

Baseline characteristics were compared using standardized mean differences rather than hypothesis testing, as p-values are influenced by sample size and provide limited information about balance quality. For outcome analyses, we calculated risk and odds ratios with 95% confidence intervals to quantify the magnitude of association.

Time-to-event analyses employed Kaplan-Meier methodology to estimate survival curves, with log-rank tests evaluating statistical significance. Cox proportional hazards regression estimated hazard ratios and confidence intervals, quantifying the relative hazard of outcomes in cohorts with elevated versus low CRP levels. The proportional hazards assumption was evaluated graphically and through statistical testing. Statistical significance was established at alpha = 0.05 (two-tailed).

## Results

### Baseline Characteristics

Prior to propensity matching, the elevated CRP cohort comprised 601,398 participants, while the low CRP cohort included 240,742 participants. Following propensity score matching, 240,727 participants remained in each group. Standardized mean differences for all matched variables were < 0.05, indicating excellent balance achievement ( Table 1 ). This balance provides confidence that observed differences in outcomes reflect CRP status rather than confounding variables.

**Table 1:** Baseline Characteristics of Propensity-Matched Cohorts Stratified by C-Reactive Protein Levels

Characteristic	Elevated CRP ( *N* = 240,727)	Low CRP ( *N* = 240,727)	*P* -value	SMD
Demographics				
Age (years), mean ± SD	64.1 ± 15.1	64.2 ± 14.9	0.375	0.003
Female	112,776 (46.8%)	111,580 (46.4%)	0.001	0.010
Male	122,748 (51.0%)	124,273 (51.6%)	< 0.001	0.013
Unknown/Not reported sex	5,203 (2.2%)	4,874 (2.0%)		
Race				
White	178,460 (74.1%)	175,403 (72.9%)	< 0.001	0.029
Black or African American	23,887 (9.9%)	24,032 (10.0%)	0.485	0.002
Asian	5,363 (2.2%)	6,290 (2.6%)	< 0.001	0.025
Other/Unknown	33,017 (13.8%)	35,002 (14.5%)	-	-
Ethnicity				
Hispanic or Latino	14,533 (6.0%)	16,009 (6.7%)	< 0.001	0.025
Not Hispanic or Latino	188,752 (78.4%)	186,268 (77.4%)	< 0.001	0.025
Unknown	37,442 (15.6%)	38,450 (16.0%)	< 0.001	0.011
Comorbidities				
Kidney disease	44,963 (18.7%)	45,458 (18.9%)	0.068	0.005
Metabolic disorders	170,632 (70.9%)	170,335 (70.8%)	0.346	0.003
Hypertensive diseases	152,294 (63.3%)	152,827 (63.5%)	0.111	0.005

### Primary Outcome: All-Cause Mortality

All-cause mortality occurred in 27,060 participants (11.3%) in the elevated CRP cohort compared with 16,782 participants (7.0%) in the low CRP cohort, yielding a risk ratio of 1.616 (95% CI 1.586–1.646; *p* < 0.001). Kaplan-Meier survival analysis demonstrated persistently lower survival probability throughout the five-year follow-up period among participants with elevated CRP. Five-year survival was 83.2% in the elevated CRP group versus 89.4% in the low CRP group.

### Secondary Cardiovascular Outcomes

OSA participants with elevated CRP demonstrated significantly higher rates across multiple cardiovascular endpoints ( Table 2 ). Cardiogenic shock—representing the most severe cardiovascular complication—affected 2,391 participants (1.0%) in the elevated CRP cohort versus 1,251 participants (0.5%) in the low CRP cohort (HR 2.003, 95% CI 1.871–2.145; *p* < 0.001), representing a doubling of risk.

**Table 2:** Primary and Secondary Outcomes in Propensity-Matched Cohorts: Five-Year Follow-up Analysis

Outcome	Elevated CRP ( *N* = 240,727)	Low CRP ( *N* = 240,727)	Risk Ratio (95% CI)	Hazard Ratio (95% CI)	P-value
Primary Outcome					
All-cause mortality	27,060 (11.3%)	16,782 (7.0%)	1.616 (1.586–1.646)	1.695 (1.662–1.727)	< 0.001
Cardiovascular Outcomes					
Cardiogenic shock	2,391 (1.0%)	1,251 (0.5%)	1.921 (1.794–2.056)	2.003 (1.871–2.145)	< 0.001
Heart failure	18,308 (9.8%)	13,213 (6.5%)	1.511 (1.479–1.544)	1.594 (1.559–1.630)	< 0.001
Acute kidney injury	22,302 (11.0%)	13,495 (6.4%)	1.720 (1.685–1.755)	1.825 (1.787–1.865)	< 0.001
Chronic kidney disease	19,034 (9.3%)	14,105 (6.9%)	1.354 (1.326–1.382)	1.433 (1.402–1.465)	< 0.001
Arrhythmic Outcomes					
Atrial fibrillation	12,400 (6.3%)	9,927 (4.8%)	1.315 (1.282–1.349)	1.372 (1.336–1.408)	< 0.001
Ventricular tachycardia	5,400 (2.3%)	4,099 (1.8%)	1.326 (1.273–1.380)	1.388 (1.332–1.445)	< 0.001
Pulmonary Outcomes					
Pulmonary hypertension	10,678 (4.9%)	7,585 (3.4%)	1.434 (1.393–1.476)	1.508 (1.464–1.553)	< 0.001
Pulmonary embolism	5,476 (2.4%)	3,831 (1.7%)	1.449 (1.391–1.509)	1.516 (1.455–1.580)	< 0.001
Other Outcomes					
Cerebrovascular disease	14,994 (7.4%)	13,372 (6.6%)	1.129 (1.104–1.155)	1.182 (1.155–1.210)	< 0.001
Pacemaker implantation	3,620 (1.5%)	2,987 (1.3%)	1.214 (1.157–1.274)	1.268 (1.208–1.331)	< 0.001

Heart failure incidence reached 18,308 participants (9.8%) in the elevated inflammatory burden group compared with 13,213 participants (6.5%) in the low inflammation group (HR 1.594, 95% CI 1.559–1.630; *p* < 0.001). This 59.4% relative risk increase underscores the substantial burden of cardiac dysfunction associated with elevated CRP in OSA.

Acute kidney injury demonstrated particularly strong association with elevated CRP status, occurring in 22,302 participants (11.0%) with elevated CRP versus 13,495 participants (6.4%) with low CRP (HR 1.825, 95% CI 1.787–1.865; *p* < 0.001). This 82.5% relative risk elevation suggests systemic effects of inflammation extending beyond cardiovascular tissues.

### Kaplan-Meier Survival Analysis

Fig. 1 illustrates Kaplan-Meier survival curves for all-cause mortality, demonstrating consistently divergent trajectories throughout the observation period. The curves separate early and maintain divergence, suggesting persistent rather than transient risk elevation. Similar patterns emerged for major cardiovascular outcomes ( Fig. 2A and 2B ), with event-free survival curves showing progressive separation over time.

**Fig. 1:**
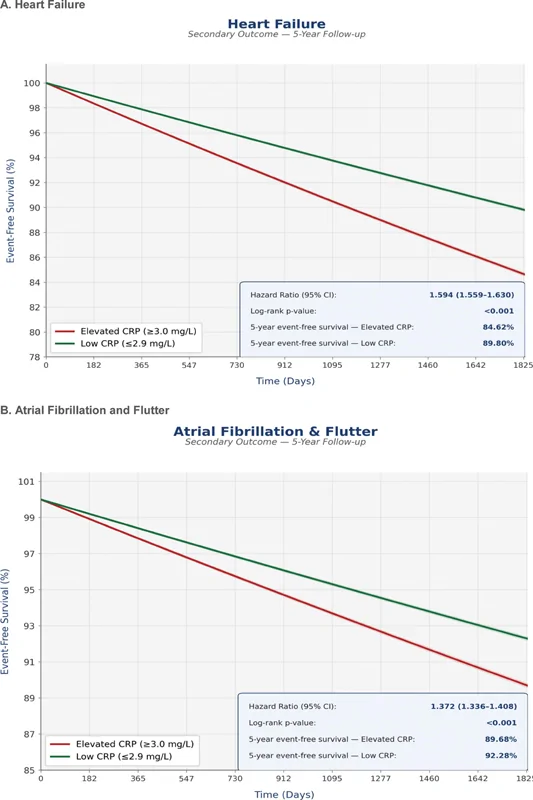
Kaplan-Meier Event-Free Survival Curves for Major Cardiovascular Outcomes. Multi-panel figure displaying event-free survival for ( **A** ) Heart Failure and ( **B** ) Atrial Fibrillation and Flutter. All panels show elevated CRP cohorts (red lines) experiencing higher event rates compared with low CRP cohorts (green lines). Hazard ratios, 95% confidence intervals, and log-rank test *p* -values are displayed for each outcome. All comparisons achieved statistical significance (p < 0.001). ( **A** ) Heart Failure: HR 1.594, 95% CI 1.559–1.630; 5–year event–free survival 84.62% versus 89.80%; p < 0.001. ( **B** ) Atrial Fibrillation and Flutter: HR 1.372, 95% CI 1.336–1.408; 5–year event–free survival 89.68% versus 92.28%; p < 0.001. Shaded bands indicate 95% confidence intervals. CRP, C-reactive protein; HR, hazard ratio; OSA, obstructive sleep apnea.

**Fig. 2:**
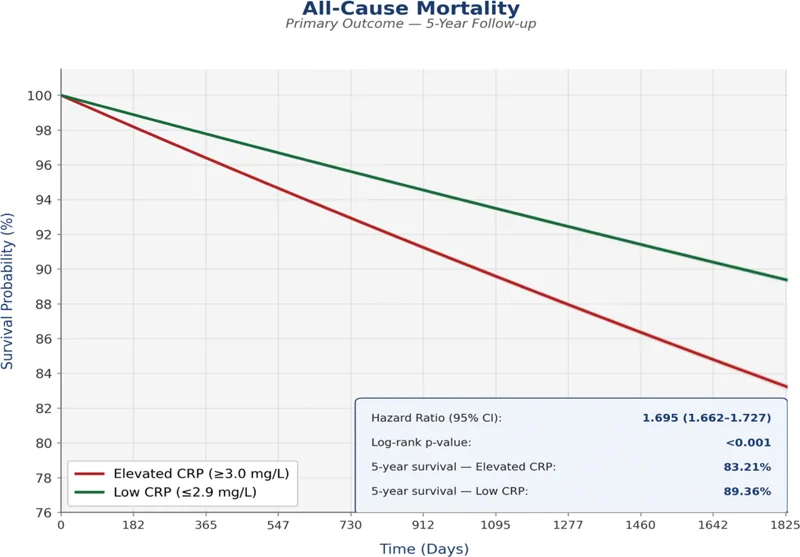
Kaplan-Meier Survival Curves for All-Cause Mortality Stratified by C-Reactive Protein Status. The graph displays five-year survival probabilities for OSA patients with elevated CRP (≥ 3.0 mg/L, red line) versus low CRP (≥ 2.9 mg/L, green line). The elevated CRP cohort demonstrated significantly lower survival throughout the follow-up period (83.21% versus 89.36% at 5 years; HR 1.695, 95% CI 1.662–1.727; log–rank p < 0.001). Shaded bands indicate 95% confidence intervals. CRP, C–reactive protein; HR, hazard ratio; OSA, obstructive sleep apnea.

## Discussion

### Principal Findings and Interpretation

This large-scale propensity-matched cohort study, encompassing nearly 500,000 OSA patients, demonstrates that elevated CRP concentrations (≥ 3.0 mg/L) strongly predict increased mortality and cardiovascular complications compared with low CRP levels. The magnitude of associations proved substantial across diverse outcomes, with mortality risk elevated by 69.5% and cardiovascular event risks increased by 37–100% depending on the specific outcome. These findings position CRP as a powerful prognostic biomarker in OSA populations, identifying individuals at highest risk for adverse outcomes who may benefit from intensified surveillance and intervention. 14

The 69.5% relative increase in all-cause mortality associated with elevated CRP is clinically significant and comparable to mortality risks conferred by other established cardiovascular risk factors. 15 For context, this magnitude of association rivals that observed for diabetes mellitus or advanced chronic kidney disease in general populations. 16 The consistency of elevated risk across multiple cardiovascular endpoints—including heart failure, arrhythmias, acute coronary syndromes, and pulmonary vascular disease—suggests that elevated CRP reflects systemic pathophysiological processes rather than isolated organ dysfunction. 17

### Mechanistic Insights: Inflammation as a Unifying Pathway

The mechanistic basis for associations between elevated CRP and adverse outcomes in OSA likely involves multiple interconnected pathways. 18 OSA-induced cyclic hypoxemia triggers hypoxia-inducible factor activation and nuclear factor-κB translocation, initiating transcription of genes encoding pro-inflammatory cytokines. 19 Interleukin-6, tumor necrosis factor-α, and interleukin-1β stimulate hepatic CRP synthesis. 20 However, CRP functions not merely as a passive marker but potentially as an active mediator of vascular injury. 21

CRP binds to oxidized low-density lipoprotein and damaged cell membranes, activating complement cascades and promoting macrophage uptake of lipoproteins—accelerating atherosclerotic plaque formation. 22 Additionally, CRP induces endothelial expression of adhesion molecules that facilitate leukocyte recruitment and perpetuate vascular inflammation, while simultaneously inhibiting endothelial nitric oxide synthase activity, thereby impairing vasodilation and contributing to endothelial dysfunction. 23 These direct vascular effects likely contribute to accelerated atherosclerosis and cardiovascular event risk observed in our elevated CRP cohort.

### Arrhythmias: Inflammation and Electrophysiological Remodeling

The elevated risk of both atrial and ventricular arrhythmias in OSA patients with high CRP warrants particular attention. Inflammation promotes arrhythmogenesis through multiple mechanisms. 24 In atrial tissue, inflammatory mediators induce structural remodeling characterized by interstitial fibrosis, which creates regions of slow conduction and facilitates reentrant circuits underlying atrial fibrillation. 25

Additionally, OSA-associated sympathetic activation interacts synergistically with inflammation to promote arrhythmias. 26 Catecholamine surges during arousal events increase automaticity and triggered activity, while inflammatory remodeling creates the substrate for sustained arrhythmias. 27 The 37-39% increased risk of atrial fibrillation and ventricular tachycardia observed in the present study suggests that CRP measurement could identify OSA patients who would benefit from enhanced arrhythmia surveillance, anticoagulation consideration, or prophylactic antiarrhythmic strategies.

### Renal Complications: Systemic Inflammation Beyond the Cardiovascular System

The particularly strong association between elevated CRP and acute kidney injury (82.5% relative risk increase) highlights the systemic nature of inflammation in OSA. Several mechanisms may contribute to this finding. OSA-induced sympathetic activation and endothelial dysfunction reduce renal perfusion, predisposing to ischemic injury. 28

The kidney injury findings also suggest potential bidirectional relationships. Chronic kidney disease promotes systemic inflammation through reduced clearance of inflammatory mediators and uremia-induced immune activation. 29 Conversely, recurrent acute kidney injury episodes accelerate chronic kidney disease progression. 30 Thus, elevated CRP in OSA patients may identify a phenotype characterized by heightened vulnerability to kidney injury, creating a vicious cycle of inflammation, renal dysfunction, and cardiovascular complications.

### Clinical Implications for Risk Stratification

Current OSA management guidelines emphasize the apnea-hypopnea index (AHI) as a measure of severity for risk stratification and treatment decisions. 31 The present findings suggest that CRP measurement could enhance risk stratification by identifying OSA patients with a heightened inflammatory burden and, correspondingly, elevated cardiovascular risk. A stratified management approach might involve: (1) routine CRP measurement at OSA diagnosis, (2) intensive cardiovascular risk factor modification for elevated CRP patients, including aggressive blood pressure control (target < 130/80 mm Hg), lipid management (LDL-C < 70 mg/dL), glycemic control in diabetics (HbA1c < 7%), and smoking cessation, (3) consideration of antiplatelet therapy in appropriate candidates, (4) enhanced monitoring for arrhythmias through periodic electrocardiography or ambulatory monitoring, and (5) potentially more aggressive CPAP adherence support and follow-up.

### Therapeutic Implications: Beyond CPAP

Continuous positive airway pressure therapy remains the cornerstone of OSA management. 32 Multiple investigations have demonstrated that effective CPAP therapy reduces CRP levels, with greater reductions observed in patients with higher baseline inflammation.33 The present findings support the importance of optimizing CPAP adherence, particularly in patients with elevated inflammatory markers, as effective therapy may reduce both CRP concentrations and associated cardiovascular risk.

However, CPAP adherence remains suboptimal in many patients, with long-term adherence rates often below 50%. 34 For patients with elevated CRP who cannot tolerate or refuse CPAP, alternative or adjunctive strategies merit consideration. Weight loss, when achievable, reduces both OSA severity and systemic inflammation. 35 Emerging therapies including hypoglossal nerve stimulation show promise for CPAP-intolerant patients. 36

### CRP Dynamics and Temporal Considerations

The present study employed a single CRP measurement at the index date, which represents a limitation but also reflects clinical practice realities. CRP concentrations demonstrate temporal variability influenced by acute illnesses, infections, injuries, and other stressors. 37 Serial CRP measurements may yield more stable risk estimates by averaging over temporal fluctuations. Some evidence suggests that CRP trajectories (increasing, stable, or decreasing over time) may provide prognostic information beyond single measurements. 38

In patients with treated OSA, CRP dynamics may reflect treatment effectiveness. Patients demonstrating CRP reductions with CPAP therapy may experience greater cardiovascular benefit than those with persistently elevated CRP despite therapy. 33 Future investigations should evaluate whether CRP monitoring during OSA treatment provides actionable information to optimize treatment.

The timing of CRP measurement relative to acute events also merits consideration. High-sensitivity CRP assays measured in stable outpatients may provide more reliable risk stratification than conventional CRP assays or measurements during acute illness. 39

### Study Limitations

Several limitations warrant acknowledgment. First, as an observational study, we cannot establish causality despite robust propensity matching. Residual confounding from unmeasured variables is an inherent constraint. Most critically, OSA severity (apnea-hypopnea index), oxygen desaturation indices, CPAP adherence, and lifestyle factors (physical activity, diet, smoking) were unavailable for adjustment. The absence of AHI and desaturation data precludes determination of whether CRP-outcome associations are independent of sleep-disordered breathing intensity—a key question for future prospective or polysomnography-linked studies. With respect to CPAP adherence, long-term compliance commonly falls below 60% in clinical populations and may be lower among patients with elevated CRP, who often carry greater metabolic and symptomatic burden. 40 If CPAP non-adherence is more prevalent in the elevated-CRP cohort, residual confounding could partially account for the observed cardiovascular risk excess, rather than inflammation alone. Similarly, lifestyle factors including physical inactivity, poor diet, and smoking each independently elevate both CRP and cardiovascular risk; their absence from the propensity model may bias estimates away from the null. 41 Randomized trials would be required to establish whether CRP-guided interventions improve outcomes.

Second, we utilized a single CRP measurement rather than serial assessments. CRP is an acute-phase reactant, and values obtained during intercurrent infections, acute illnesses, or inflammatory flares may not reflect the chronic low-grade inflammatory burden relevant to cardiovascular risk in OSA. 7 The TriNetX platform does not provide the clinical context of each laboratory draw in a format that permits exclusion of measurements obtained during acute illness; accordingly, patients with transiently elevated CRP may have been misclassified into the elevated-CRP cohort. Such misclassification would likely be non-differential with respect to outcomes, biasing estimates toward the null rather than inflating effect sizes. Beyond measurement context, CRP variability over time may further attenuate observed associations, and CRP trajectories may carry additional prognostic value beyond a single time point. High-sensitivity CRP assays obtained under standardized, fasting, stable-state conditions would provide more reliable risk stratification in future prospective studies.

Third, outcome ascertainment relied on ICD-10 diagnostic codes, which may be subject to misclassification. However, validated coding algorithms demonstrate good accuracy for the outcomes examined, and non-differential misclassification would bias results toward the null. The consistency of findings across diverse endpoints reduces the likelihood that coding error accounts for the observed associations.

Fourth, the study population derived from TriNetX-participating healthcare systems and may not fully represent all OSA patients. Selection bias could occur if patients with more severe disease or better healthcare access are preferentially included; however, the network's breadth across 130 institutions enhances generalizability relative to single-center studies.

Fifth, the five-year follow-up period, while substantial, may be insufficient to capture the full long-term cardiovascular consequences of chronic inflammation. Inflammation status may also change over time with treatment, potentially attenuating initial risk differences during extended observation.

### Future Research Directions

Several research priorities emerge from our findings. First, randomized controlled trials should evaluate whether CRP-guided treatment strategies improve outcomes in OSA. Such trials might compare standard care with intensified interventions (aggressive risk factor modification, anti-inflammatory therapies, enhanced monitoring) in patients with OSA and elevated CRP. Demonstration of outcome benefits would be necessary to justify routine clinical implementation of CRP-based risk stratification.

Second, investigations should determine optimal CRP thresholds for risk classification in OSA populations. Although we employed the 3.0 mg/L threshold per cardiovascular guidelines, OSA-specific thresholds may differ. Receiver operating characteristic analyses can identify CRP cut points that maximize sensitivity and specificity for adverse outcomes. Additionally, continuous CRP modeling might reveal non-linear dose–response relationships.

Third, studies should evaluate whether CPAP therapy and other OSA interventions reduce CRP levels and whether CRP reductions mediate cardiovascular benefits. Demonstrating that effective OSA treatment lowers inflammation and that inflammation reduction explains improved outcomes would strengthen causal inference. Serial CRP measurements in treated patients could identify those requiring additional anti-inflammatory interventions.

Fourth, investigations should assess whether combining CRP with other biomarkers enhances risk prediction. Multimarker approaches incorporating inflammatory markers, cardiac biomarkers (natriuretic peptides, troponin), and metabolic markers might provide superior discrimination compared with CRP alone. Machine learning algorithms integrating diverse biomarkers with clinical variables could optimize risk stratification.

Fifth, mechanistic studies should clarify pathways linking inflammation to cardiovascular complications in OSA. Experimental models could evaluate whether CRP directly mediates vascular injury or serves primarily as a biomarker of upstream inflammatory processes. Understanding causal pathways would inform therapeutic target selection and guide intervention development.

Finally, implementation science research should address practical questions about integrating CRP measurement into clinical workflows. Optimal timing and frequency of CRP assessment, cost-effectiveness of CRP-guided strategies, and patient perspectives on biomarker-based risk stratification all require evaluation before widespread clinical adoption.

## Conclusion

This large-scale propensity-matched analysis demonstrates that elevated CRP concentrations strongly predict mortality and cardiovascular complications in OSA patients. **The magnitude and consistency of associations across diverse outcomes are consistent with CRP serving as a marker of clinically significant inflammatory burden in OSA; whether this association reflects a causal contribution of CRP to adverse outcomes cannot be determined from the present observational design and requires confirmation in prospective or experimental studies.** These findings support consideration of inflammatory biomarker assessment as a component of comprehensive cardiovascular risk stratification in OSA populations.

While additional research is needed to establish clinical utility and cost-effectiveness, CRP measurement represents a readily available, inexpensive, and standardized biomarker that could enhance identification of high-risk OSA patients. Such patients might benefit from intensified monitoring, aggressive cardiovascular risk factor modification, and optimization of OSA-specific therapies. Ultimately, integrating inflammatory biomarkers into OSA care algorithms may facilitate personalized medicine approaches that match intervention intensity to individual risk profiles, improving outcomes while optimizing resource utilization.

## References

[1] Iannella G Pace A Bellizzi M G The Global Burden of Obstructive Sleep Apnea Diagnostics (Basel) 2025 15 09 1088 10.3390/diagnostics15091088 PMC12071658 40361906

[2] Pham L V Schwartz A R The pathogenesis of obstructive sleep apnea J Thorac Dis 2015 7 08 1358 1372 10.3978/j.issn.2072-1439.2015.07.28 PMC4561284 26380762

[3] Liu L Wang Y Hong L Bragazzi N L Dai H Chen H Obstructive Sleep Apnea and Hypertensive Heart Disease: From Pathophysiology to Therapeutics Rev Cardiovasc Med 2023 24 12 342 10.31083/j.rcm2412342 PMC11272867 39077075

[4] Griendling K K Camargo L L Rios F J Alves-Lopes R Montezano A C Touyz R M Oxidative Stress and Hypertension Circ Res 2021 128 07 993 1020 10.1161/CIRCRESAHA.121.318063 PMC8293920 33793335

[5] Zhili G Zhimin G Nenghua Z Role of vascular endothelial dysfunction and metabolic reprogramming of immune cells in the development and progression of atherosclerosis Front Physiol 2025 16 1.624225E6 10.3389/fphys.2025.1624225 PMC12441067 40969916

[6] Fiedorczuk P Polecka A Walasek M Olszewska E Potential Diagnostic and Monitoring Biomarkers of Obstructive Sleep Apnea-Umbrella Review of Meta-Analyses J Clin Med 2022 12 01 60 10.3390/jcm12010060 PMC9821668 36614858

[7] Sproston N R Ashworth J J Role of C-Reactive Protein at Sites of Inflammation and Infection Front Immunol 2018 9 754 10.3389/fimmu.2018.00754 PMC5908901 29706967

[8] Ahtesham M Khan S W Khan S R C-reactive Protein (CRP) Levels as a Predictor of Adverse Cardiovascular Events in Acute Myocardial Infarction: A Prospective Study Cureus 2025 17 10 e93943 10.7759/cureus.93943 PMC12588365 41200636

[9] Shamsuzzaman A S Winnicki M Lanfranchi P Elevated C-reactive protein in patients with obstructive sleep apnea Circulation 2002 105 21 2462 2464 10.1161/01.cir.0000018948.95175.03 12034649

[10] Ninić A Rajkov B Kotur-Stevuljević J Association Between Redox and Inflammatory Biomarkers with the Presence and Severity of Obstructive Sleep Apnea Medicina (Kaunas) 2025 61 09 1557 10.3390/medicina61091557 PMC12471577 41010948

[11] Manzur A R Negru A G Florescu A R Obstructive Sleep Apnea and Outcomes in Cardiac Surgery: A Systematic Review with Meta-Analytic Synthesis (PROSPERO CRD420251049574) Biomedicines 2025 13 07 1579 10.3390/biomedicines13071579 PMC12292749 40722654

[12] Khosla A A Nasir K Saxena A Association between high risk of obstructive sleep apnea and inflammatory markers in a population sample of young and middle-aged adults in the Miami Heart Study J Clin Sleep Med 2024 20 12 1895 1903 10.5664/jcsm.11274 PMC11609831 38975989

[13] Singh B Goyal A Patel B C C-Reactive Protein: Clinical Relevance and Interpretation. [Updated 2025 May 3] Treasure Island (FL) StatPearls Publishing 2025Jan-. Available from:https://www.ncbi.nlm.nih.gov/books/NBK441843/28722873

[14] Huang T Goodman M Li X C-reactive Protein and Risk of OSA in Four US Cohorts Chest 2021 159 06 2439 2448 10.1016/j.chest.2021.01.060 PMC8213962 33529772

[15] Bouloukaki I Mermigkis C Kallergis E M Moniaki V Mauroudi E Schiza S E Obstructive sleep apnea syndrome and cardiovascular disease: The influence of C-reactive protein World J Exp Med 2015 5 02 77 83 10.5493/wjem.v5.i2.77 PMC4436942 25992322

[16] Afkarian M Sachs M C Kestenbaum B Kidney disease and increased mortality risk in type 2 diabetes J Am Soc Nephrol 2013 24 02 302 308 10.1681/ASN.2012070718 PMC3559486 23362314

[17] Zhou H H Tang Y L Xu T H Cheng B C-reactive protein: structure, function, regulation, and role in clinical diseases Front Immunol 2024 15 1.425168E6 10.3389/fimmu.2024.1425168 PMC11211361 38947332

[18] Yi M Zhao W Tan Y The causal relationships between obstructive sleep apnea and elevated CRP and TNF-α protein levels Ann Med 2022 54 01 1578 1589 10.1080/07853890.2022.2081873 PMC9176672 35652886

[19] Htoo A K Greenberg H Tongia S Activation of nuclear factor kappaB in obstructive sleep apnea: a pathway leading to systemic inflammation Sleep Breath 2006 10 01 43 50 10.1007/s11325-005-0046-6 16491391

[20] Tanaka T Narazaki M Kishimoto T IL-6 in inflammation, immunity, and disease Cold Spring Harb Perspect Biol 2014 6 10 a016295 10.1101/cshperspect.a016295 PMC4176007 25190079

[21] Ansar W Ghosh S.Inflammation and Inflammatory Diseases, Markers, and Mediators: Role of CRP in Some Inflammatory Diseases. Biology of C Reactive Protein in Health and Disease.2016Mar 24:67–107.

[22] Singh S K Suresh M V Voleti B Agrawal A The connection between C-reactive protein and atherosclerosis Ann Med 2008 40 02 110 120 10.1080/07853890701749225 PMC3364506 18293141

[23] Hein T W Singh U Vasquez-Vivar J Devaraj S Kuo L Jialal I Human C-reactive protein induces endothelial dysfunction and uncoupling of eNOS in vivo Atherosclerosis 2009 206 01 61 68 10.1016/j.atherosclerosis.2009.02.002 PMC2735606 19268941

[24] Bi X Zhang S Jiang H Mechanistic insights into inflammation-induced arrhythmias: a simulation study Front Physiol 2022 13 843292 10.3389/fphys.2022.843292 PMC9196871 35711306

[25] Scott L Jr Li N Dobrev D Role of inflammatory signaling in atrial fibrillation Int J Cardiol 2019 287 195 200 10.1016/j.ijcard.2018.10.020 PMC6447485 30316645

[26] May A M Van Wagoner D R Mehra R OSA and Cardiac Arrhythmogenesis: Mechanistic Insights Chest 2017 151 01 225 241 10.1016/j.chest.2016.09.014 PMC5989643 27693594

[27] Birză M R Negru A G Frent Ș M New Insights of Cardiac Arrhythmias Associated with Sleep-Disordered Breathing: From Mechanisms to Clinical Implications-A Narrative Review J Clin Med 2025 14 06 1922 10.3390/jcm14061922 PMC11942725 40142730

[28] Abuyassin B Sharma K Ayas N T Laher I Obstructive Sleep Apnea and Kidney Disease: A Potential Bidirectional Relationship? J Clin Sleep Med 2015 11 08 915 924 10.5664/jcsm.4946 PMC4513269 25845900

[29] Kadatane S P Satariano M Massey M Mongan K Raina R The Role of Inflammation in CKD Cells 2023 12 12 1581 10.3390/cells12121581 PMC10296717 37371050

[30] Venkatachalam M A Griffin K A Lan R Geng H Saikumar P Bidani A K Acute kidney injury: a springboard for progression in chronic kidney disease Am J Physiol Renal Physiol 2010 298 05 F1078 F1094 10.1152/ajprenal.00017.2010 PMC2867413 20200097

[31] Malhotra A Ayappa I Ayas N Metrics of sleep apnea severity: beyond the apnea-hypopnea index Sleep 2021 44 07 zsab030 10.1093/sleep/zsab030 PMC8271129 33693939

[32] Luong T V Hoang T A Tran D H Continuous positive airway pressure therapy for patients with obstructive sleep apnea and coronary artery disease World J Cardiol 2025 17 10 107750 10.4330/wjc.v17.i10.107750 PMC12576564 41181658

[33] Zhu Q Luo Q Wang Z Chen S Chen G Huang S Effects of continuous positive airway pressure therapy on inflammatory markers in patients with obstructive sleep apnea: a meta-analysis of randomized controlled trials Sleep Breath 2025 29 02 182 10.1007/s11325-025-03348-6 PMC12064446 40346316

[34] Catcheside P G Predictors of continuous positive airway pressure adherence F1000 Med Rep 2010 2 70 10.3410/M2-70 PMC2954420 20948830

[35] López-Padrós C Salord N Alves C Effectiveness of an intensive weight-loss program for severe OSA in patients undergoing CPAP treatment: a randomized controlled trial J Clin Sleep Med 2020 16 04 503 514 10.5664/jcsm.8252 PMC7161448 32003737

[36] Hernandez L Trelles-Garcia D Medina M Sarna K Smolley L Hadeh A Effectiveness of Hypoglossal Nerve Stimulation Therapy in Positional Obstructive Sleep Apnea: A Retrospective Observational Study J Clin Med 2025 14 16 5873 10.3390/jcm14165873 PMC12387958 40869698

[37] Cherny S S Brzezinski R Y Wasserman A Characterizing CRP dynamics during acute infections Infection 2025 53 03 1199 1203 10.1007/s15010-024-02422-7 PMC12137512 39467995

[38] Ke J Liu Q Liu X Prognostic value of C-reactive protein predicting all-cause and cause-specific mortality: a prospective cohort study in Shanghai, China BMJ Open 2025 15 08 e101532 10.1136/bmjopen-2025-101532 PMC12352246 40803722

[39] Banait T Wanjari A Danade V Banait S Jain J Role of High-Sensitivity C-reactive Protein (Hs-CRP) in Non-communicable Diseases: A Review Cureus 2022 14 10 e30225 10.7759/cureus.30225 PMC9650935 36381804

[40] Rotenberg B W Murariu D Pang K P Trends in CPAP adherence over twenty years of data collection: a flattened curve J Otolaryngol Head Neck Surg 2016 45 01 43 10.1186/s40463-016-0156-0 PMC4992257 27542595

[41] Islam M A Morshed R Alam S Hossain J Islam M S Association Between Lifestyle and Dietary Habits With C-Reactive Protein and Glucose: A Cross-Sectional Study Health Sci Rep 2026 9 01 e71741 10.1002/hsr2.71741 PMC12794028 41531539

